# Dr. Brown’s Dental Depot

**Published:** 1867-03

**Authors:** 


### DR. BROWN'S DENTAL DEPOT.

"John Brown's body lies mouldering in the grave;
But his *soul* is marching on:"

And J. M. Brown's Dental Depot stands towering, corner of Fourth and Walnut, and *its soul is marching on*, and has been ever since the body took up its present locality, in "the good old days" when a cobbler's awl was a standard excavator, and a tailor's bodkin an approved plugger. Then, and for long years afterward, Dr. Brown kept the only Dental Depot in the great Mississippi Valley. It was the *sole* depot, and he the *soul* of it. It and *he* have marched to the music of professional progress ever since; or the profession has marched to the tune of the Doctor's progress, we don't know which. They were together then, and are together now. When *any thing* is wanted, call on J. M, Brown, or *send him word that you want it;* and it will come.

